# BandQuant: a web application for western blot quantification

**DOI:** 10.1093/bioadv/vbag292

**Published:** 2026-10-05

**Authors:** Muyang He, Shanglin Jin, Hongjian Wan, Chengfeng Zhang

**Affiliations:** Department of Dermatology, Huashan Hospital, Fudan University, Shanghai 200040, China; Department of Dermatology, Huashan Hospital, Fudan University, Shanghai 200040, China; Shanghai Art-Coding Software Co., Ltd., Shanghai 200052, China; Department of Dermatology, Huashan Hospital, Fudan University, Shanghai 200040, China

## Abstract

**Motivation:**

Western blot quantification typically requires multiple tools and manual data handling, which is time-consuming and difficult to audit. Existing software is either tied to proprietary hardware or requires local installation, and none provides an integrated path from raw image to fold-change.

**Results:**

We present BandQuant, a free, browser-based web application that integrates the entire western blot quantification workflow into a single session. It combines automated band detection, rolling-ball background subtraction, housekeeping normalization, fold-change computation, and interactive bar-chart visualization in a single interface. A relational database binds experimental metadata to the image at upload, enabling automated normalization and cross-experiment queries without manual relabeling. BandQuant measurements showed high agreement with Fiji (Pearson *r* = 0.994), and analysis time was reduced by 65%.

**Availability and implementation:**

BandQuant is freely available at https://www.tiaodaibao.com/? locale=en-US. Source code: https://github.com/wanhj-git/BandQuant. Archived release: https://zenodo.org/records/19453998.

## 1 Introduction

Western blotting is the most widely used method for protein detection and quantification in biomedical research, with over 400 000 publications indexed in PubMed referencing the technique (Towbin *et al.* 1979, Bass *et al.* 2017, Gomes 2023). Through electrophoresis and membrane transfer, proteins are separated by molecular weight and transferred to a solid-phase membrane. Antibody-based immunodetection then selectively labels the target protein, and enhanced chemiluminescence (ECL) or fluorescence imaging produces the final band images, whose size and intensity indicate target protein quantity. To calibrate protein content with cell counts, housekeeping genes such as GAPDH and β-actin are employed as normalization factors. Fold-change values are then calculated against a control group to quantify experimental effects.

To achieve normalization, band intensity must first be converted from pixel grayscale values into quantitative numeric data (Gassmann *et al.* 2009, Taylor *et al.* 2013). ImageJ, including its Fiji distribution, provides flexible ROI measurement, lane-profile analysis, and an extensive plugin ecosystem (Schindelin *et al.* 2012, Schneider *et al.* 2012, Schroeder *et al.* 2021). However, downstream normalization and reporting generally require separate data handling.

Image Lab (Bio-Rad Laboratories) and iBright Analysis Software (Thermo Fisher Scientific) integrate automated lane and band detection, background correction and normalization with their respective imaging workflows; iBright also offers desktop and cloud access. These systems streamline instrument-centered analysis, but group-level statistics and cross-experiment queries are outside their documented standard workflows.

IOCBIO Gel (Kütt *et al.* 2023) prioritizes traceability by storing the original image, analysis steps, and metadata in a relational database, with results available through export or database queries. GelBox (Gulbulak *et al.* 2024) uses image preprocessing and Gaussian curve fitting to quantify closely spaced or overlapping bands and stores images, metadata, and analyses in a single file. Both are open source, but require local Python or MATLAB environments and do not combine loading-control normalization, replicate statistics, and fold-change visualization within their standard workflows.

BandQuant complements these approaches as a free, open-source browser application that links images to sample and target metadata and integrates band detection, loading-control normalization, replicate statistics, fold-change plots, and cross-experiment queries. The principal workflow features are summarized in Table 1.

**Table 1:** Comparison of BandQuant with existing densitometry tools.^a^

Feature	ImageJ/Fiji	Image Lab	iBright	IOCBIO Gel	GelBox	BandQuant
**Web access**	No	No	Yes^b^	No	No	Yes
**Open source**	Yes	No	No	Yes	Yes	Yes
**Auto band detection**	No	Yes	Yes	No	No	Yes
**Full-lane profiles**	Yes	Yes	Yes	Yes	No^c^	Yes
**Built-in normalization**	No	Yes	Yes	No^d^	No	Yes
**Replicate statistics**	No	No	No	No	No	Yes
**Per-target loading controls**	No	No	No	No	No	Yes
**Cross-experiment query**	No	No	No	Yes^e^	No	Yes

a Yes indicates availability within the documented standard workflow; functions requiring exported data, custom scripts, or third-party plugins are recorded as No.

b iBright has cloud and desktop versions.

c GelBox analyses profiles within manually defined ROIs.

d IOCBIO Gel requires Excel or SQL for within-lane target-to-loading-control ratios.

e IOCBIO Gel cross-experiment queries require SQL.

## 2 Availability and implementation

BandQuant is accessible at https://www.tiaodaibao.com/?locale=en-US and is compatible with all major browsers; no additional installation is required. Source code is available at https://github.com/wanhj-git/BandQuant; an archived release is deposited at https://doi.org/10.5281/zenodo.19453997. A complete workflow and data architecture diagram is provided in Fig. S1, available as supplementary data at *Bioinformatics Advances* online.

BandQuant is implemented as a client–server web application. The frontend is built with Ant Design Pro, the backend with Node.js and Express, and data are stored in a MySQL database. Image processing is performed server-side using Python. All data are encrypted in transit and at rest and are accessible only to the registered account holder. A registration-free “Speed Mode” is also available, in which users may upload images and download quantification results without any data being retained on the server.

## 3 BandQuant

At the core of BandQuant is a relational data model (experiment → sample → target protein → band) that reflects the biological structure of western blot experiments. All outputs (raw intensity, normalized values, and fold-change) inherit this hierarchy, so the same target can be retrieved or compared across experimental batches without re-annotation. This structure is reflected in the data dashboard and in every downstream computation described below (Fig. 1).

**Figure 1:**
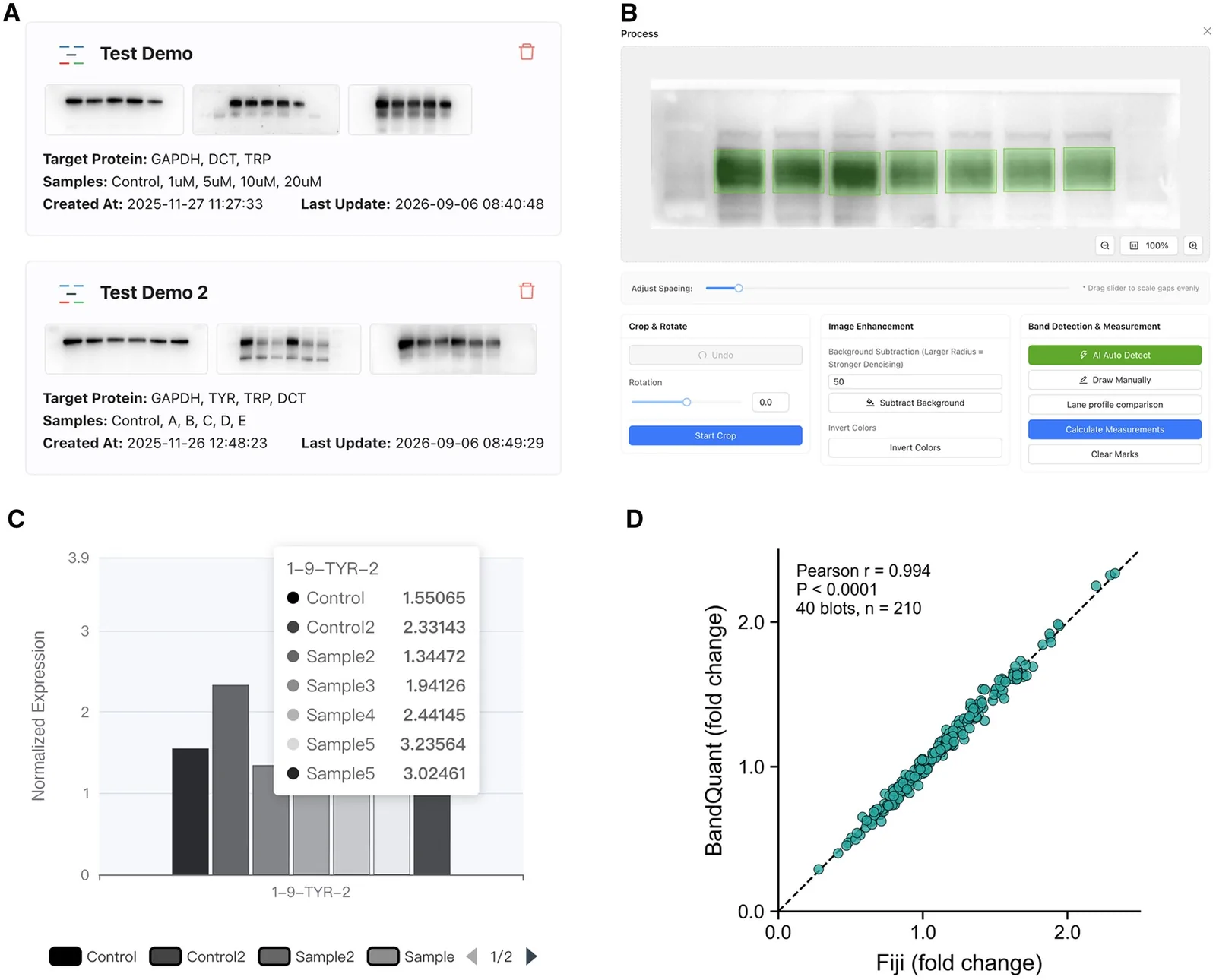
Overview of BandQuant. (A) Project dashboard with band thumbnails. (B) Interactive band-extraction interface. (C) Quantification chart panel, with exact values displayed on hover. (D) Agreement between Fiji and BandQuant across 210 non-control measurements from 40 western blot images (Pearson *r* = 0.994, *P* < .0001). Abbreviation: A.U., arbitrary units.

Upon login, the user enters the data dashboard (Fig. 1A). Saved experiments are displayed as cards showing band thumbnails and annotations including target proteins, samples, and creation time. A folder sidebar on the left allows multi-level experiment categorization, and a search bar in the upper right enables filtering by various metadata fields.

Selecting “New Experiment” initiates the main workflow. The user first enters the experiment name, then enters or imports lane information, including sample names, sample groups, control designation, and sample volume. Control samples can be individually designated. When a single membrane contains multiple target proteins, lane-level metadata are inherited across all targets without re-entry, and each target can be assigned its own loading control. The user then uploads band images and labels each with its corresponding protein name. Cropping is available to remove regions outside the bands of interest upon upload.

The core quantification is performed in the “Data Processing” panel. The left subpanel offers background signal subtraction based on the rolling-ball algorithm (Sternberg 1983), so that inter-sample differences are accurately reflected. Users can adjust the radius of the rolling-ball algorithm to control smoothing strength (default 50 px). Image polarity inversion in the middle subpanel converts bright-background images to a dark-background representation so that darker pixels correspond to higher signal values. The right subpanel is for selecting band regions of interest (ROIs) on each lane. BandQuant renders an interactive bounding-box overlay on the band image, showing the region used for intensity calculation (Fig. 1B). Initial bounding boxes are generated automatically using morphological filtering and adaptive thresholding. Users can also refine box positions and sizes through a spacing-adjustment slider and drag-and-drop repositioning. All boxes are of the same size by default. Finally, the integrated density of each box is computed (integrated density = mean gray value × area), matching the standard ImageJ measurement (Schneider *et al.* 2012), and mapped accordingly. Alternatively, users can select a complete lane for intensity-profile analysis and compare it with rectangular-ROI quantification without altering the ROI-based results. BandQuant then performs control-based normalization, fold-change calculation, and group-level statistical analysis. Users may export results as CSV for local analysis.

As real-world samples often contain different amounts of total protein, pilot experiments frequently produce uneven housekeeping-protein expression across lanes, complicating trend interpretation. In such cases, “Reference Alignment” calculates, based on the housekeeping-protein signal and a user-designated reference sample, the adjusted loading volume required for the remaining samples to match the reference in total protein content.

Finally, the “Chart” panel offers interactive bar chart visualization of three parameters: raw signal intensity, normalized (reference-corrected) values, and fold-change values. Charts are available in color and grayscale styles and are downloadable as .png files (Fig. 1C). A composite figure tool assembles band images alongside sample and protein name labels in a grid layout.

In addition, a registration-free “Speed Mode” provides the same quantification workflow with samples identified by numerical indices; results can be downloaded but are not retained server-side.

To validate quantification accuracy, we obtained 210 non-control measurements from 40 western blot images using Fiji and BandQuant. The images spanned a range of exposure conditions and band-to-background contrasts. The measurements showed high agreement (Pearson *r* = 0.994, *P* < .0001; Fig. 1D). BandQuant also substantially increased processing efficiency. In a benchmark on two images each containing 6 lanes, BandQuant completed the full workflow in ∼1.5 min, compared with ∼4 min for the equivalent Fiji workflow (a 65% reduction in processing time). This advantage becomes more pronounced when dealing with multiple targets, as repeated manual selection and data formatting are saved.

## 4 Discussion

BandQuant consolidates western blot densitometric analysis and structured data management into a single, free web application. By binding experimental metadata to the image at upload, it enables automated normalization, lane mapping, and visualization without external spreadsheets. BandQuant showed high agreement with Fiji while substantially reducing analysis time, and its persistent record from raw image to fold-change improves the reproducibility of western blot quantification (Gassmann *et al.* 2009) in line with FAIR principles (Wilkinson *et al.* 2016).

BandQuant has several limitations. Its rectangular ROIs and integrated-density measurement can be less accurate when band profiles partially overlap (Gulbulak *et al.* 2024), and curved-lane geometries are not supported (Kütt *et al.* 2023). Total-protein normalization (e.g. stain-free imaging; Aldridge *et al.* 2008, Eaton *et al.* 2013) is also not yet supported. Future work will evaluate deep-learning–based band segmentation (Meijering 2020), alternative background-correction algorithms (Gulbulak *et al.* 2024), exportable analysis packages for sharing with collaborators or reviewers, a fully local installable version for laboratories handling sensitive data, and extension to other gel-based assays such as northern blots.

## Supplementary Material

vbag292_Supplementary_Data

## Data Availability

The data underlying this article are available in the BandQuant GitHub repository at https://github.com/wanhj-git/BandQuant and in Zenodo at https://doi.org/10.5281/zenodo.19453997. Demo datasets used for validation and benchmarking are provided in the /demo directory of the repository.
